# Comparative assessment of CacyBP/SIP, β‐catenin and cannabinoid receptors in the adrenals of hypertensive rats

**DOI:** 10.1111/jcmm.18376

**Published:** 2024-05-23

**Authors:** Magdalena Smereczańska, Natalia Domian, Alicja Lewandowska, Irena Kasacka

**Affiliations:** ^1^ Department of Histology and Cytophysiology Medical University of Bialystok Bialystok Poland

**Keywords:** adrenal glands, CacyBP/SIP, CB1, CB2, hypertension, β‐Catenin

## Abstract

Taking into account homeostatic disorders resulting from arterial hypertension and the key importance of CacyBP/SIP, β‐catenin and endocannabinoids in the functioning of many organs, it was decided to assess the presence and distribution of CacyBP/SIP, β‐catenin, CB1 and CB2 in the adrenal glands of hypertensive rats of various aetiology. The study was conducted on the adrenal glands of rats with spontaneous and renovascular hypertension. The expression of CacyBP/SIP, β‐catenin, CB1 and CB2 was detected by immunohistochemistry and real‐time PCR method. The results of the present study revealed both lower gene expression and immunoreactivity of CacyBP/SIP in the adrenal glands of all hypertensive groups compared to the normotensive rats. This study demonstrated a reduction in the immunoreactivity and expression of the β‐catenin, CB1 and CB2 genes in the adrenals of 2K1C rats. While in SHR, the reaction showing β‐catenin and CB1 was very weak or negative, and the expression of CB2 in the adrenal glands of these rats increased. The results of this study show, for the first time, marked differences in the expression of CacyBP/SIP, β‐catenin and CB1 and CB2 cannabinoid receptors in the adrenal glands of rats with primary (SHR) and secondary hypertension (2K1C).

## 1 INTRODUCTION

Hypertension is one of the leading risk factors for heart disease and stroke, the leading causes of death worldwide. Untreated high blood pressure leads to many dangerous consequences. They are associated primarily with damage to blood vessels, which in turn leads to organ dysfunction.

Based on the detectability and reversibility of the direct cause of this disease, two types of hypertension are distinguished. Primary hypertension, which is noted in approximately 85%–90% of patients suffering from hypertension. In this type, the direct cause of the disease is not identifiable. Secondary hypertension, with distribution 5%–10% of patients with hypertension, is characterised by the ability to determine the direct cause of hypertension; moreover, this cause can be reversed. The most common culprits of secondary hypertension are renovascular disease and renal parenchymal disease. In addition to high blood pressure values, progressive renal failure and the manifestation of central nervous system symptoms, there are disorders of the functions of other organs, including the adrenal glands.^1^, ^2^ Hypertension is accompanied by abnormal secretion of both mineralocorticosteroids (involved in the regulation of electrolyte balance) and catecholamines that affect cardiovascular homeostasis.^1^, ^2^ Despite the growing knowledge about the pathogenesis of hypertension and its treatment, the organ complications mentioned above are the dominant cause of death in the world.^3^

CacyBP/SIP is a small, multifunctional protein discovered in the 1990s. Previous studies have shown that it is involved in the processes of dephosphorylation, degradation of β‐catenin, reorganisation of the cytoskeleton, ubiquitination and stress response in the cell.^4^

β‐catenin is a structural component of cell junctions, binding E‐cadherin with the cytoskeleton, and after being released from the cell membrane into the cytoplasm, it becomes a link of the WNT transmitter pathway.^5^ Canonical Wnt signalling relies on the accumulation of the multifunctional β‐catenin protein, which is involved in both the regulation of transcription and the interaction of E‐cadherins with cytoskeleton proteins.^6^ The Wnt/β‐catenin pathway can also be examined in the context of primary aldosteronism (PA), a disease that is one form of secondary hypertension.^7^ PA arises from the autonomic production of aldosterone in the adrenal glands, which causes hypertension with an increased ratio of aldosterone/renin and hypokalaemia.^8^ Diagnosis of secondary hypertension is important both in the therapy of the primal cause of the disease and in the prevention of cardiovascular complications.

One of the basic physiological systems of the human body is the endocannabinoid system (ECS). It plays the role of the guardian of many processes taking place in the body. It is made up of CB1 and CB2 receptors spread throughout the body. These receptors are influenced by endocannabinoids produced by the body. The endogenous cannabinoid system also includes enzymes that synthesise and break down endocannabinoids. Cannabinoids exert potent antihypertensive and cardioprotective effects through complex mechanisms involving direct and indirect effects on the heart muscle and vascular system.^9^, ^10^ On the one hand, endocannabinoids and cannabinoid receptors are involved in the hypotensive state associated, inter alia, with hemorrhagic shock, and on the other hand, play an important role in regulating the circulatory system in hypertension.^9^

The ECS also reduces the activity of stress pathways, including the hypothalamic–pituitary–adrenal (HPA) pathways. The HPA axis is one of the main conductors of the endocrine system, which sends messages to the body through hormones. Adrenal hormones are primarily responsible for regulating stress, energy, adrenaline and blood pressure.^10^ Endocannabinoids affect hemodynamic parameters of the circulatory system as well as oxidative stress and inflammatory changes, which are responsible for the progression of hypertension. Furthermore, endocannabinoids might influence the regulation of the blood pressure by reducing the secretion of aldosterone.^11^

The proteins we selected are involved in the inflammatory process underlying hypertension. Hypertension is believed to be a chronic inflammatory disease. Organ complications may be related not only to hemodynamic factors but also to increased levels of inflammatory markers. In particular, CB1 and CB2 receptors can modulate the immune response in the inflammatory process. CacyBP/SIP and β‐catenin influence inflammation‐related processes, although their direct relationship to this process is still not well documented. The aim of our research was to assess the expression of the studied parameters in an organ exposed to hypertension of various aetiologies and to check whether the differences would be greater in the case of one of these types of hypertension.

The interaction of CacyBP/SIP with S100 family proteins is well documented, which depends on the concentration of Ca^2+^ ions and occurs at physiological concentrations of this cation. It cannot be ruled out that such interactions, dependent on the concentration of calcium ions, also occur between CacyBP/SIP and other proteins. So far, the function of CacyBP complexes with other proteins has not been clearly determined. However, it is suggested that S100A6, through interaction with CacyBP/SIP, may inhibit the activity of the ubiquitin ligase Siah‐1‐CacyBP/SIP‐Skp1‐TBL1 (SCF^TBL1^) and thus affect the level of β‐catenin protein, which has many important functions in cardiovascular system. There is a close relationship between β‐catenin and hypertension. Disturbances of the Wnt/β‐catenin pathway lead to impaired functioning of organs directly related to the regulation of blood pressure. Intracellular calcium content is regulated by cannabinoids, which modulate the flow of calcium ions through membrane channels. Endocannabinoids, acting through CB1 and CB2 receptors, regulate blood pressure, heart rate and heart contractility. Perhaps cannabinoid receptors regulate the intracellular content of Ca^2+^ ions and influence the interactions between various protein complexes involved in signalling pathways related to hypertension, including the proteins we studied. Taking into account homeostatic disorders resulting from arterial hypertension and the key importance of CacyBP/SIP, β‐catenin and endocannabinoids in the functioning of many organs, the question arises, whether and to what extent the type of hypertension affects the expression of tested parameters in the adrenal glands. The aim of this study was to comparatively evaluate the expression and localization of CacyBP/SIP and β‐catenin proteins, as well as CB1 and CB2 receptors in the adrenal glands of rats with arterial hypertension of various aetiologies.

## 2 MATERIALS AND METHODS

### 2.1 Experimental animals

The assumptions, the aim and the plan of the study, as well as the approach to animals, were approved by the local Ethics Committee for Studies on Animal Subjects in Białystok.

The study was performed on five (5) normotensive male Wistar Kyoto rats (WKY), seven (7) male spontaneously hypertensive rats (SHR) and 12 (*n* = 12) young male Wistar rats. Their body weight at the beginning of the experiment was within 180–200 g. The animals were kept in lighted and ventilated conditions with room temperature and maintained day and night rhythm. The rats had free access to standard granulated chow and a normal drinking water.

Male spontaneously hypertensive rats were purchased from Polish Mother's Memorial Hospital Research Institute in Łódź, Poland.

The experimental animals were divided into six groups:

**Table:** 

SHR	Seven (7) rats with genetically determined systemic hypertension, inbred strain established from Wistar rats selected for high blood pressure
WKY	Five (5) normotensive Wistar Kyoto rats, being the reference for SHR rats
2K1C	Seven (7) Wistar rats with renovascular hypertension induced by ligation of the artery supplying the blood to the left kidney (two kidney, one clip model of hypertension)
Sham‐operated	Five (5) Wistar rats underwent sham operation (submitted to the same surgical procedure as the hypertensive rats, however, without arterial ligation), being the reference for 2K1C

### 2.2 2K1C renovascular hypertension

Induction of experimental hypertension was performed according to procedure by Goldblatt et al.^12^ After the rats were anaesthetized by exposure to pentobarbital (40 mg/kg, i.p.), a 3‐cm retroperitoneal flank incision was performed under sterile conditions. The left kidney was exposed, and the renal artery was carefully dissected free of the renal vein. The renal artery was then partially occluded by placing a silver clip with an internal diameter of 0.20 mm on the vessel. The wound was closed with a running 3–0 silk suture (*n* = 10). Sham‐operated rats (*n* = 5) underwent identical surgical procedures except that a clip was not applied to the renal artery. After the surgery, the rats were kept in single cages until wound healing. Normotensive control rats were submitted to the same surgical procedure as the hypertensive rats, however, without arterial ligation (underwent sham operation).

### 2.3 Blood pressure measurement

The systolic BP was measured in all wakeful animals by using a non‐invasive tail‐cuff method. BP measurements were considered valid only when three consecutive readings did not differ by more than 5 mmHg. The measurements of BP proved the systolic hypertension in SHR and 2K1C rats (those animals had values of SBP equal to or higher than 160 mmHg).

### 2.4 Method of experimental material collection and fixation

At 6 weeks of experiment, the fragments of adrenals were collected under deep pentobarbital anaesthesia (50 mg/kg of body weight) from all rats. Obtained adrenal tissues were immediately fixed in 10% buffered formalin solution and routinely embedded in paraffin or placed in RNA‐later solution (AM7024 Thermo Fischer) and stored at −80°C.

Adrenal paraffin blocks were cut into section of 4 μm thickness and then stained with haematoxylin‐eosin for general histological examination and processed by immunohistochemistry to detect CacyBP/SIP, β‐catenin, CB1 and CB2. Material stored in RNA‐later solution was processed by real‐time PCR to evaluate the expression of genes coding CacyBP/SIP, β‐catenin, CB1 and CB2.

### 2.5 Identification of CacyBP/SIP, β‐catenin, CB1 and CB2 by immunohistochemical method

In the immunohistochemical study, the EnVision method was used, as previously described by Kasacka et al.^13^ Immunohistochemistry was performed using an REAL™ EnVision™ Detection System, Peroxidase/DAB, Rabbit/Mouse detection kit (K5007; Dako Cytomation). Immunostaining was performed by the following protocol. Paraffin‐embedded sections were deparaffined and hydrated in pure alcohols. For antigen retrieval, the sections were subjected to pre‐treatment in a pressure chamber heated for 1 min at 144.7 kPa at 125°C. During antigen retrieval sections for detection of CacyBP/SIP, β‐catenin, CB1 and CB2 were incubated with Target Retrieval Solution Citrate pH = 6.0 (S 2369 Dako Cytomation). After cooling down to room temperature, the sections were incubated with Peroxidase Blocking Reagent (S 2023 Dako Cytomation) for 5 min to block endogenous peroxidase activity. Subsequently, the sections were incubated with the primary antibody for CacyBP/SIP (rabbit polyclonal antibody to CacyBP/SIP, ab190950 Abcam), β‐catenin (rabbit monoclonal antibody to β‐catenin, ab32572 Abcam), CB1 (rabbit polyclonal antibody to CB1, ab137410 Abcam) and CB2 (rabbit polyclonal antibody to CB2, ab3561 Abcam). The antisera were previously diluted in antibody diluent (S 0809 Dako Cytomation) in relation 1:600 for CacyBP/SIP, 1:2000 for β‐catenin, 1:800 for CB1 and 1:200 for CB2. Incubation with primary antibodies lasted 24 h and was carried out at 4°C in a humidified chamber. Procedure was followed by incubation (1 h) with secondary antibody (dextran coupled with peroxidise molecules and goat secondary antibody molecules against rabbit and mouse immunoglobulins) (Dako REAL™ EnVision™/ HRP Rabbit/Mouse (ENV) K50071 Agilent DGG Polska). The bound antibodies were visualised by 1‐min incubation with dako REAL™ DAB+ chromogen. The sections were finally counterstained in haematoxylin QS (H – 3404, Vector Laboratories; Burlingame, CA), mounted and evaluated under light microscope. Appropriate washing with Wash Buffer (S 3006 Dako Cytomation) was performed between each step (3 times for 5 min). Sections were dehydrated with absolute alcohol, followed by xylene and coverslipped with Entellan (Merck).

Specificity tests performed for the CacyBP/SIP, β‐catenin, CB1 and CB2 antibody included: negative control, where the primary antibodies were omitted and only antibody diluent was used, and a positive control was prepared with specific tissue as it was recommended by the manufacturer. Histological preparations were evaluated using an Olympus BX43 light microscope (Olympus 114 Corp., Tokyo, Japan) with an Olympus DP12 digital camera (Olympus 114 Corp., Tokyo, Japan) and documented.

### 2.6 Real‐time PCR

Samples of adrenal gland were taken from each rat and placed in an RNA‐later solution. Total RNA was isolated using NucleoSpin® RNA Isolation Kit (Machery‐Nagel). Quantification and quality control of total RNA were determined using the spectrophotometer – NanoDrop 2000 (ThermoScientific, Waltham, MA, USA). Only RNA samples for which the absorbance ratio at wavelength 260 nm/280 nm was 1.8–2.1 were adopted for the next analysis steps. The mentioned absorbance ratio shows that the isolated RNA was not contaminated with protein. An aliquot of 1 μg of total RNA was reverse transcribed into cDNA using iScript™ Advanced cDNA Synthesis Kit for RT‐qPCR (BIO‐RAD, Barkley, California, USA). Synthesis of cDNA was performed in a final volume of 20 μL using a thermal cycler (Model SureCycler 8800, Aligent Technologies). For reverse transcription, the mixtures were incubated at 46°C for 20 min, then heated to 95°C for 1 min and finally rapidly cooled at 4°C.

Quantitative real‐time PCR reactions were performed using Stratagene Mx3005P (Aligent Technologies) with the SsoAdvanced™ Universal SYBER® Green Supermix (BIO‐RAD, Barkley, California, USA). Specific primers for the CacyBP/SIP (*Cacybp*), β‐catenin (*Ctnnb1*), CB1 (*Cnr1*), CB2 (*Cnr2*) and GAPDH (*Gapdh*) were designed by BIO‐RAD Company. The housekeeping gene GAPDH (*Gapdh*) was used as the reference gene for quantification. In order to determine the amounts of tested genes expression levels, standard curves for each gene were constructed separately with serially diluted PCR products. PCR products were obtained by amplification of cDNA using specific primers as follows: *Cacybp* (qRnoCED0006717, BIO‐RAD), *Ctnnb1* (qRnoCID0053256, BIO‐RAD), *Cnr1* (qRnoCED0008430, BIO‐RAD), *Cnr2* (qRnoCED0008595, BIO‐RAD) and *Gapdh* (qRnoCID0057018, BIO‐RAD) were carried out in a duplicate in a final volume of 20 μL under the following conditions: 2 min polymerase activation at 95°C, 5 s denaturation at 95°C, 30 s annealing at 60°C for 35 cycles. PCR reactions were checked by including no‐RT controls, by omission of templates and by melting curve to ensure only a single product was amplified. The relative quantification of gene expression was determined by comparison of values of *C*_t_ using the ΔΔ*C*_t_ method. All results were normalised to *Gapdh*.

### 2.7 Quantitative analysis

From each animal, 12 sections of adrenal were studied (three sections for each: CacyBP/SIP, β‐catenin, CB1 and CB2 immunostaining). Five randomly selected microscopic fields [each field 0.785 mm^2^, 200× magnification (20× lens and 10× eyepiece)] from each adrenal section were documented using an Olympus DP12 microscope camera. Each digital image of the adrenal section was morphometrically evaluated using NIS Elements AR 3.10 Nikon for microscopic image analysis.

The intensity of the immunohistochemical reaction for CacyBP/SIP, β‐catenin, CB1 and CB2 was measured in each image and determined using a grey scale level of 0–255, where the value of the completely white or light pixel is 0, while the completely black pixel is 255.

All collected data were statistically analysed by means of software computer package Statistica Version 13.3. The corresponding mean values were calculated automatically; due to the lack of normality of the distribution of the obtained results, a non‐parametric Kruskal–Wallis test was performed to assess the statistical significance of differences between the groups (WKY/SHR and SHAM/2K1C). *p* < 0.05 was taken as the level of significance.

In order to analyse the correlation between the tested proteins, statistically calculated *r* ^2^, the regression equation and the correlation coefficient. The results of this analysis are shown as the *β* coefficient (this metric represents the percentage of the dependent variable's change for each unit of the independent variable's change), *r* ^2^ (shows the percentage of one variable that is responsible for the variability of the other) and the statistical significance (*p*). The relationship between two variables was acknowledged to be statistically significant at the value of the *β* coefficient for which *p* < 0.05.

## 3 RESULTS

Systolic hypertension was found in all rats in the studied groups (SHR and 2K1C).

**FIGURE 1:**
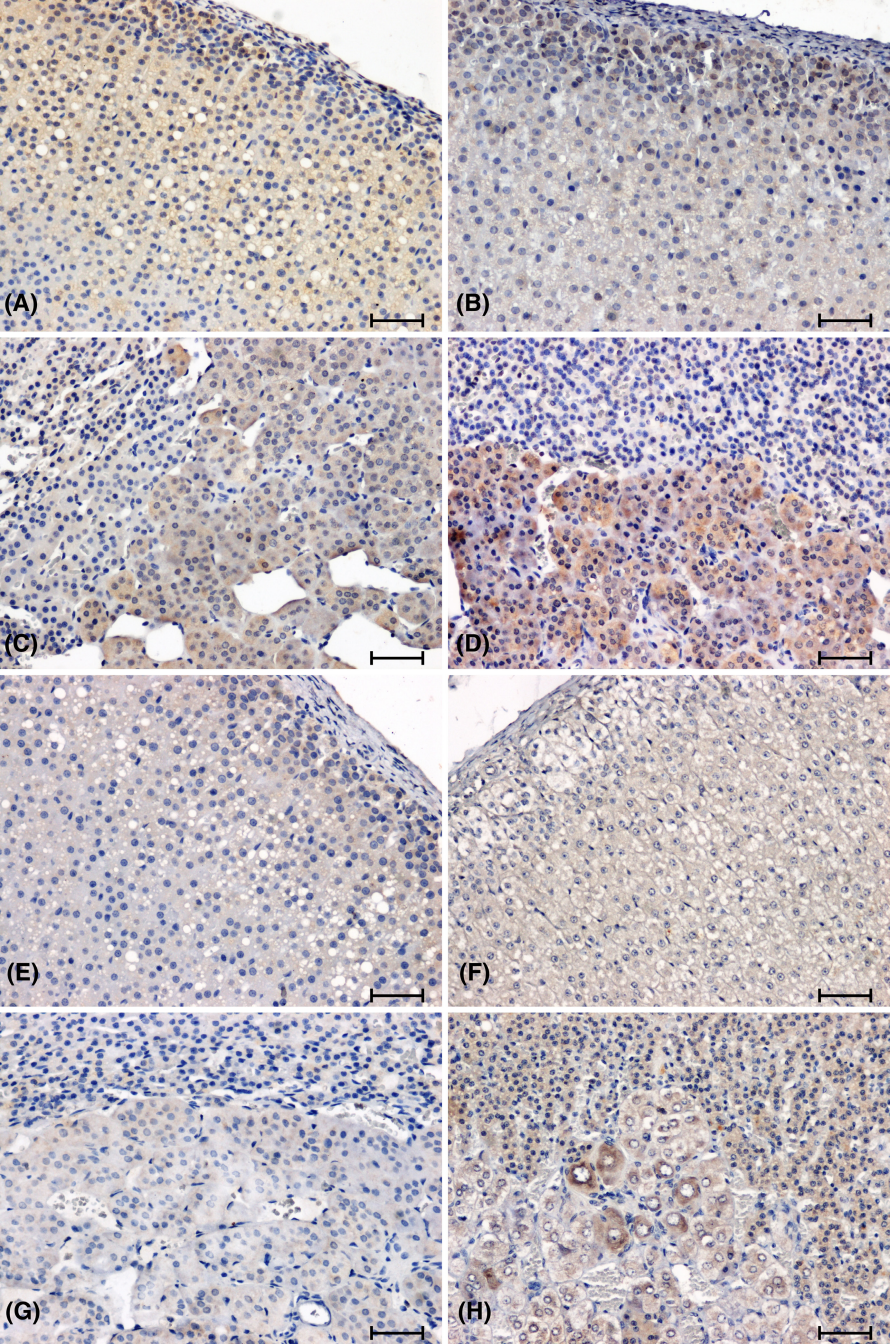
Immunodetection of CacyBP/SIP in adrenal glands of normotensive (A–D) and hypertensive (E–H) rats. Adrenal cortex and medulla of WKY (A, C), sham‐operated rat (B, D); Adrenal cortex and medulla of SHR (E, G); 2K1C rat (F, H). Scale bar = 50 μm.

The immunostaining of CacyBP/SIP in the adrenal glands of normotensive animals produced a notable reaction in cortex and medulla (Figure 1A–D). The intensity of the CacyBP/SIP immunolabelling was higher in the adrenal cortex of the WKY rats (Figure 1A) compared to the rats of the remaining control group (Figure 1B). In the adrenal medulla of control rats, a weaker reaction was seen in WKY rats (Figure 1C). In the adrenal glands of hypertensive rats (Figure 1E–H), a reduction in immunoreactivity against CacyBP/SIP was found compared to normotensive animals (Figure 1A–D). Whereas the weakest reaction in the adrenal cortex of animals with hypertension was observed in the group of 2K1C rats (Figure 1F). In the adrenal medulla of SHR rats, the CacyBP/SIP‐immunoreaction was very weak (Figure 1G), while in the group of rats with renovascular hypertension, it was clearly visible in some cells (Figure 1H).

**FIGURE 2:**
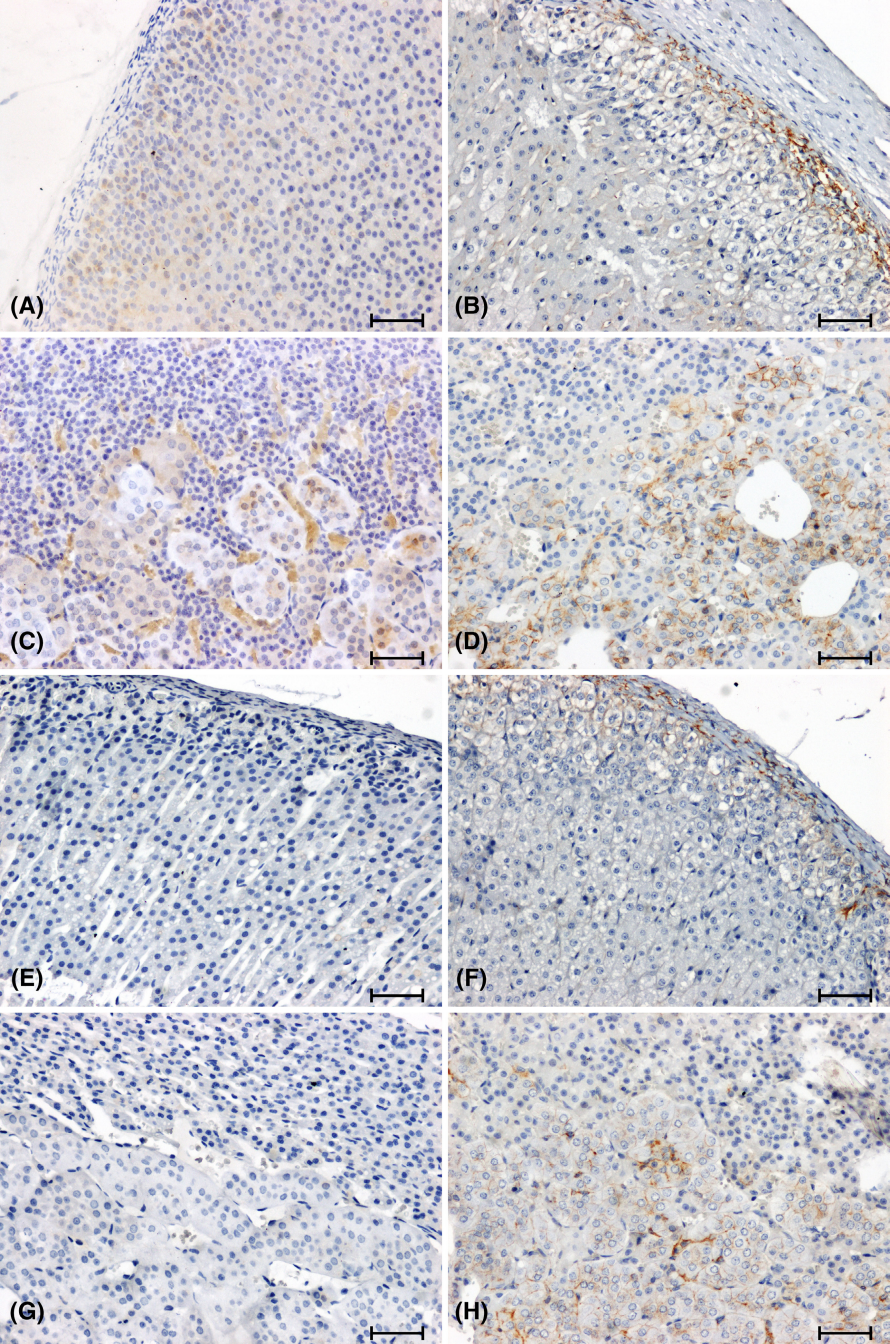
Immunohistochemical reaction determining β‐catenin in adrenal glands of normotensive (A–D) and hypertensive (E–H) rats. Adrenal cortex and medulla of WKY rat (A, C), sham‐operated (B, D); Adrenal cortex and medulla of SHR rat (E, G); 2K1C (F, H). Scale bar = 50 μm.

The anti‐β‐catenin antibody showed a weak reaction in the cortex and a moderate response in the adrenal medulla of WKY rats (Figure 2A,C), while no reaction was detected in the adrenal glands of SHR rats (Figure 2E,G). In the adrenal glands of the 2K1C (Figure 2F,H) rats, the intensity of the β‐catenin immunoreaction was weakened compared to the corresponding control group (Figure 2B,D). In the tested hypertension models, the intensity of β‐catenin immunolabelling was weak in the 2K1C group (Figure 2F,H) and negative in the SHR rats (Figure 2E,G).

**FIGURE 3:**
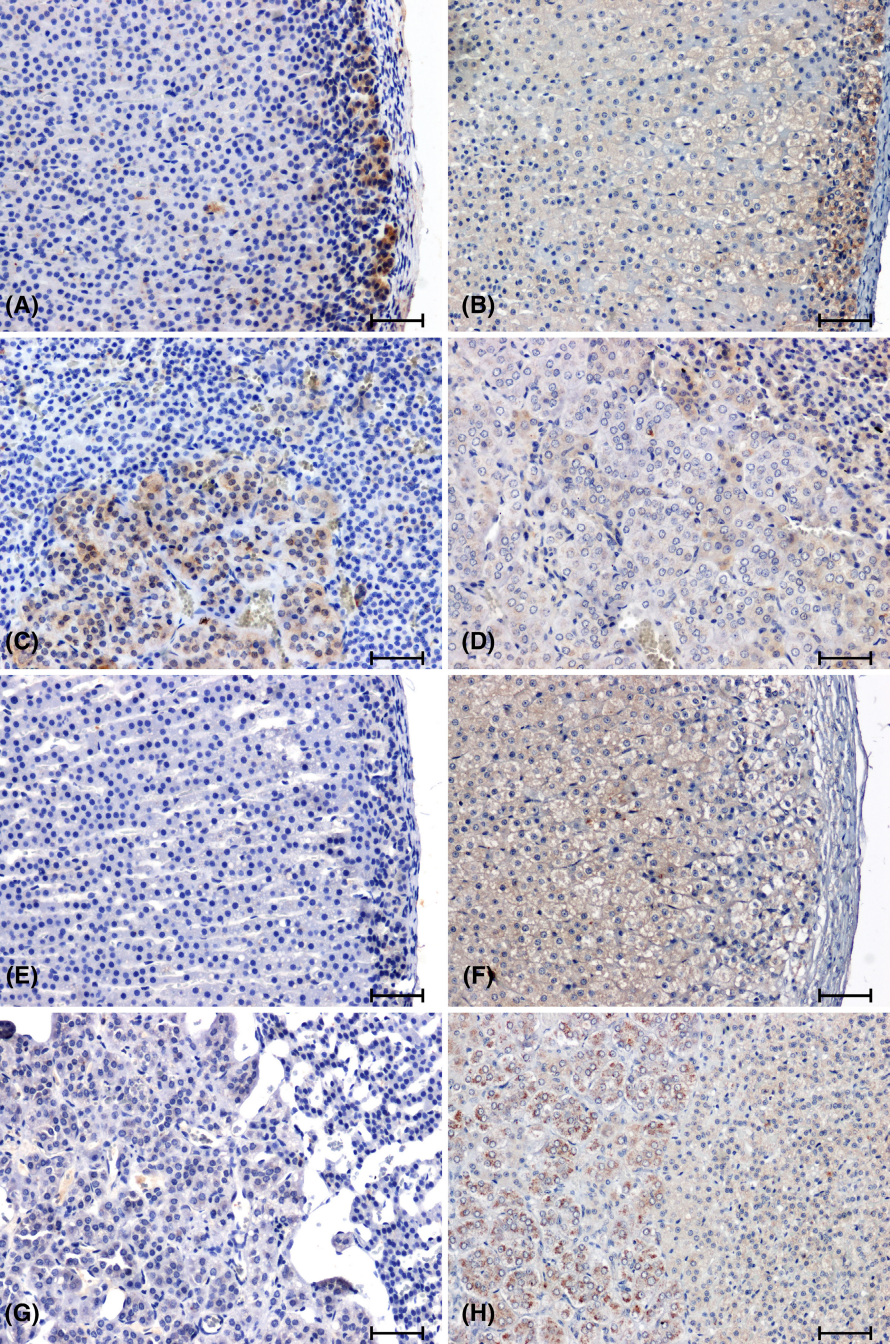
Immunolabelling of CB1 receptor in adrenal glands of normotensive (A–D) and hypertensive (E–H) rats. Adrenal cortex and medulla of WKY rat (A, C), sham operated rat (B, D); Adrenal cortex and medulla of SHR rat (E, G); 2K1C (F, H). Scale bar = 50 μm.

Strong CB1 immunoreactivity was demonstrated in the glomerular layer of the cortex of WKY rats (Figure 3A). In animals in this control group, the strongest CB1‐immunoreactivity was also noted in the adrenal medulla (Figure 3C). In all the hypertensive rats, the response to the presence of CB1 was reduced (Figure 3F,H) or negative (Figure 3E,G) compared to the corresponding control group (Figure 3A–D).

**FIGURE 4:**
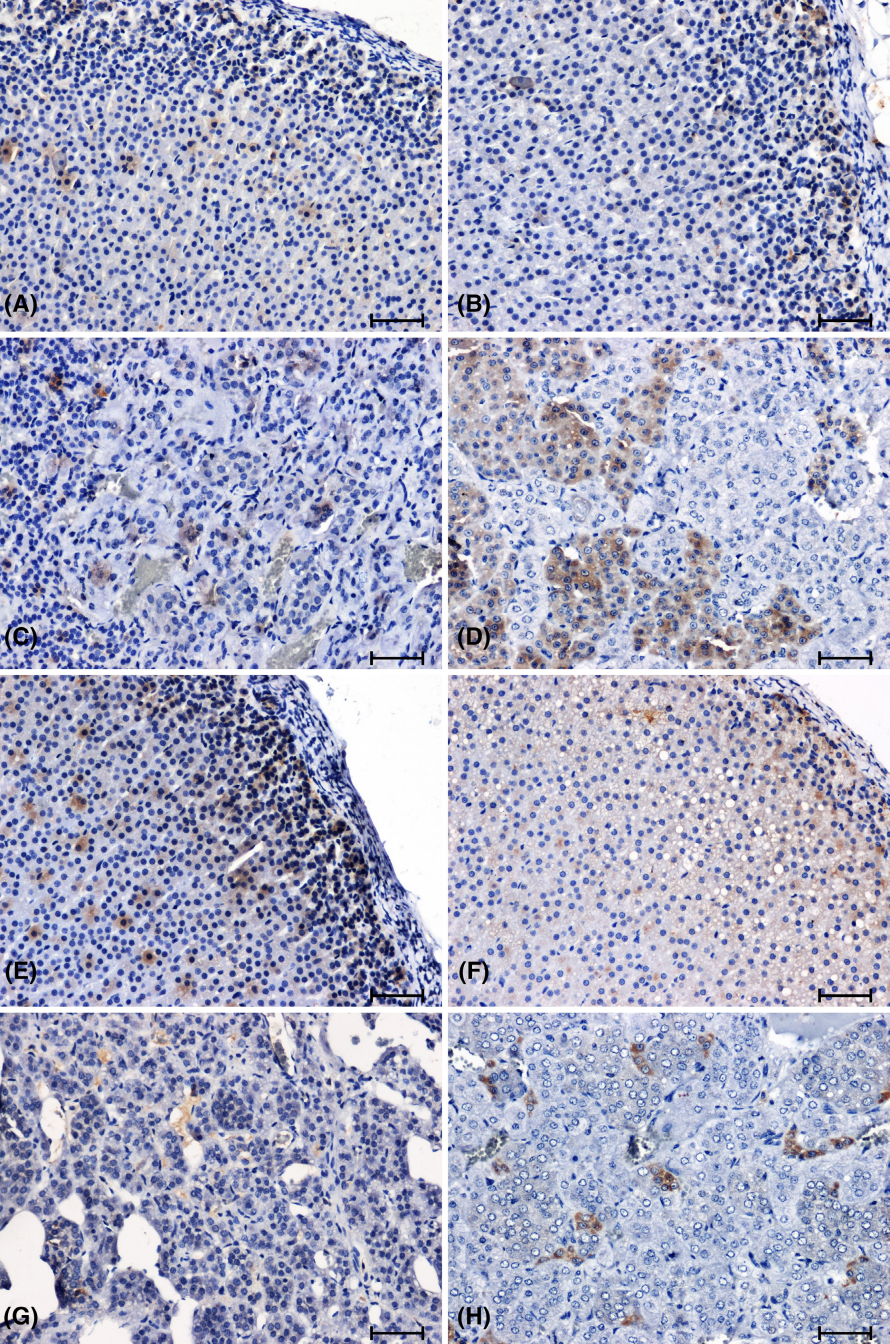
Immunohistochemical reaction determing CB2 receptor in adrenal glands of normotensive (A–D) and hypertensive (E–H) rats. Adrenal cortex and medulla of WKY rat (A, C), sham‐operated rat (B, D); Adrenal cortex and medulla of SHR rat (E, G); 2K1C rat (F, H). Scale bar = 50 μm.

In the adrenal glands of SHR rats, there was a significant increase in the CB2 immunoreaction, especially in the cortex of the organ (Figure 4E,G), compared to that observed in WKY animals (Figure 4A, C). In the 2K1C hypertensive rats (Figure 4F,H), there was a reduction in CB2 immunoreactivity relative to the corresponding control group (Figure 4B,D).

Computer image analysis confirmed visually perceived changes in the intensity of the immunohistochemical reaction against CacyBP/SIP, β‐catenin, CB1 and CB2 in the adrenal glands of hypertensive rats of various aetiologies (Table 1).

**TABLE 1:** The intensity of immunoreaction determining CacyBP/SIP, β‐catenin, CB1 and CB2 receptor in adrenals of normotensive and hypertensive rats (mean ± SE).

		Intensity of immunohistochemical reaction in rat adrenal glands Scale from 0 (white pixel) to 255 (black pixel)			
		Control groups		Hypertensive groups	
		Control 1 (WHY)	Control 2 (SHAM‐OPERATED)	SHR	2K1C
CacyBP/SIP	Zona glomerulosa of adrenal cortex	102.7 ± 3.43	85.4 ± 4.91	87.7 ± 4.58*↓	75.5 ± 3.47
	Zones fasciculata and reticularis of adrenal cortex	96.7 ± 2.61	78.8 ± 1.68	49.8 ± 1.39*↓	68.5 ± 2.03
	Adrenal medulla	101.1 ± 5.58	107.3 ± 4.99	68.7 ± 4.66*↓	105.6 ± 3.61
β‐catenin	Zona glomerulosa of adrenal cortex	77.1 ± 8.17	139.9 ± 8.90	not detected (0)*↓	136.3 ± 5.08
	Zones fasciculata and reticularis of adrenal cortex	60.4 ± 4.47	70.3 ± 4.35	not detected (0)*↓	51.8 ± 1.62*↓
	Adrenal medulla	81.1 ± 5.58	86.6 ± 7.77	not detected (0)*↓	76.7 ± 7.47
CB1	Zona glomerulosa of adrenal cortex	156.4 ± 8.40	139.2 ± 6.84	not detected (0)*↓	112.0 ± 7.97*↓
	Zones fasciculata and reticularis of adrenal cortex	90.6 ± 9.40	80.6 ± 4.21	not detected (0)*↓	63.3 ± 1.76*↓
	Adrenal medulla	132.9 ± 4.41	119.4 ± 5.14	not detected (0)*↓	108.6 ± 5.00
CB2	Zona glomerulosa of adrenal cortex	105.6 ± 5.12	108.6 ± 4.63	158.1 ± 5.34*↑	101.7 ± 7.20
	Zones fasciculata and reticularis of adrenal cortex	75.4 ± 4.19	82.6 ± 2.85	100.8 ± 3.59*↑	81.8 ± 4.26
	Adrenal medulla	87.6 ± 5.49	123.3 ± 3.75	93.7 ± 5.21	107.5 ± 4.76*↓

*Note*: ↑ refers to the intensification of immunohistochemical reaction, and ↓ refers to the weakening of immunohistochemical reaction.

* *p* < 0.05 control group versus hypertensive group.

**FIGURE 5:**
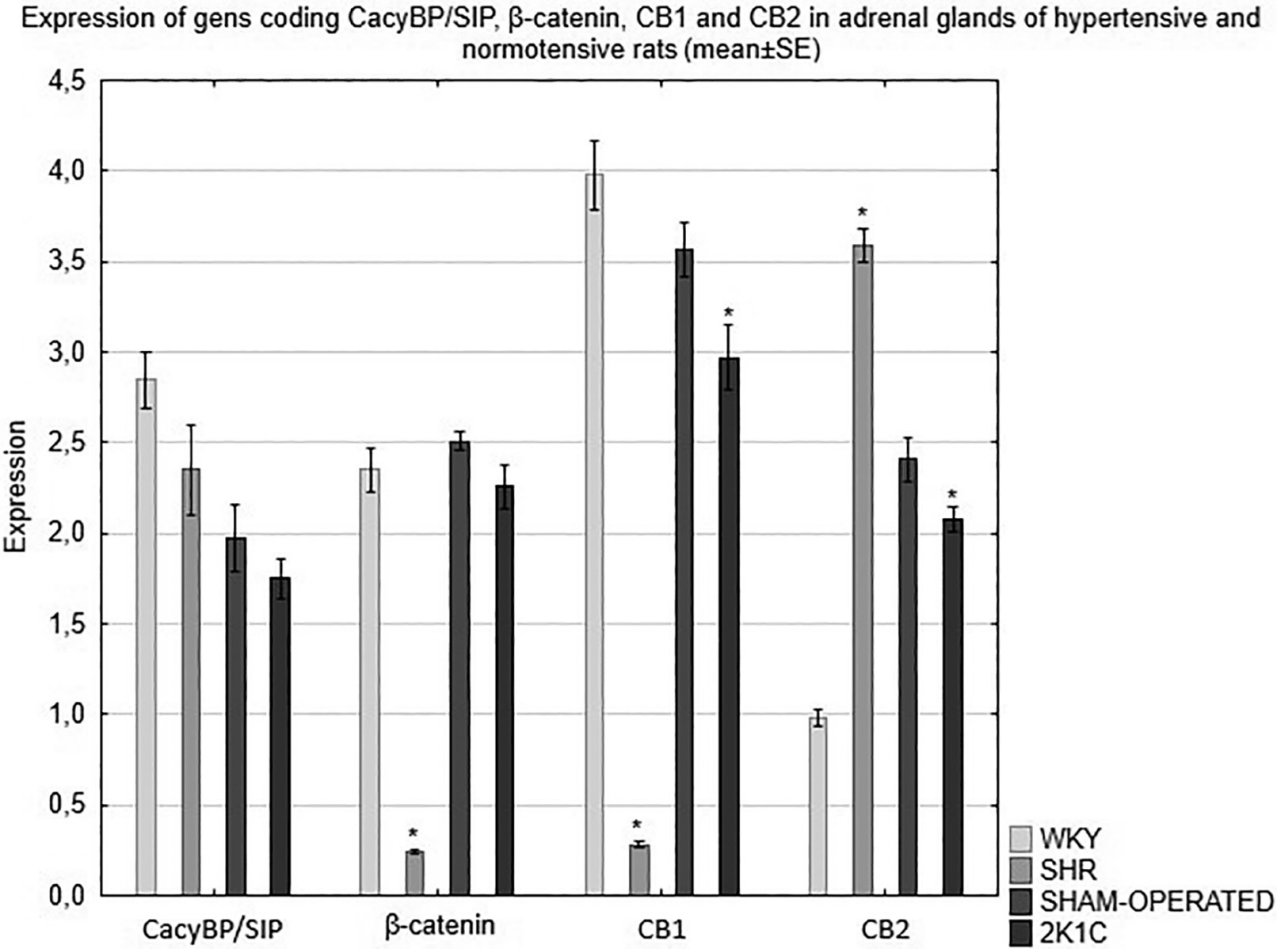
Expression of genes coding CacyBP/SIP, β‐catenin, CB1 and CB2 in the adrenal glands of normotensive and hypertensive rats. *p < 0.05 control group versus hypertensive group. Control rats (WKY and SHAM‐OPERATED) and hypertensive rats (SHR, 2K1C).

The expression of the CacyBP/SIP, β‐catenin, CB1 and CB2 genes in the adrenal glands of rats depends on the type of hypertension. RT‐qPCR analysis revealed a decrease in the expression of the CacyBP/SIP gene in the adrenal glands of all hypertensive animals compared to the corresponding control group. The analysis of the results showed a significantly weaker expression of the β‐catenin gene in the adrenal glands of the hypertensive rats compared to the respective normotensive rats. Detailed analysis of the RT‐qPCR results revealed also a significant decrease in the expression of the gene encoding CB1 in the adrenal glands of the hypertensive rats as compared to the corresponding control group. The studies showed significantly lower expression of the CB2 gene in the adrenal glands of the 2K1C rats compared to the control group. On the other hand, in the SHR model, the expression of the gene encoding CB2 was significantly higher compared to the WKY normotensive rats (Figure 5).

Statistical analysis showed co‐expression only between several parameters we examined these results were marked in red and with an asterisk * (*p* > 0.05). As for the relationships between the tested proteins, in the cortex of rats with spontaneous hypertension, mutually positive relationships were found only between CB1 and CB2 receptors. Whereas, in the adrenal medulla of SHR rats, a positive correlation was found between β‐catenin and both CBD receptors and between CB1 and CB2. Several mutual dependencies were also found between the parameters determined in the adrenal glands of rats with secondary hypertension. In the adrenal cortex of animals with this type of hypertension, a relationship between β‐catenin and the CB2 receptor was found. In the adrenal medulla of rats with secondary hypertension, a positive correlation was demonstrated between β‐catenin and cannabinoid receptors, as well as between the CB1 and CB2 receptors. There was no relationship between other parameters tested in the adrenal glands of rats with primary and secondary hypertension. The results of the correlation between the tested proteins, divided into primary and secondary hypertension, are presented in Tables 2 and 3.

**TABLE 2:** Correlation analysis between the tested proteins in the cortex and medulla of rats with spontaneous hypertension.

Spontaneous hypertension – Adrenal cortex				
	CACYBP/SIP	β‐catenin	CB1	CB2
CACYBP/SIP	–	β = +0.6498 *p* = 0.1098 *r* ^2^ = 0.1359	β = −0.2527 *p* = 0.3182 *r* ^2^ = 0.0553	β = +0.211 *p* = 0.5475 *r* ^2^ = 0.0205
β‐catenin	β = +0.6498 *p* = 0.1098 *r* ^2^ = 0.1359	–	β = −0.042 *p* = 0.7726 *r* ^2^ = 0.0048	β = +0.3692 *p* = 0.0515 *r* ^2^ = 0.1946
CB1	β = −0.2527 *p* = 0.3182 *r* ^2^ = 0.0553	β = −0.042 *p* = 0.7726 *r* ^2^ = 0.0048	–	β = +0.9399 *p* = 0.0009* *r* ^2^ = 0.4687
CB2	β = +0.211 *p* = 0.5475 *r* ^2^ = 0.0205	β = +0.3692 *p* = 0.0515 *r* ^2^ = 0.1946	β = +0.9399 *p* = 0.0009* *r* ^2^ = 0.4687	–
**Spontaneous hypertension – Adrenal medulla**				
	**CACYBP/SIP**	**β‐catenin**	**CB1**	**CB2**
CACYBP/SIP	–	β = −0.6225 *p* = 0.1480 *r* ^2^ = 0.2427	β = +0.8884 *p* = 0.2753 *r* ^2^ = 0.1463	β = +17,748 *p* = 0.1542 *r* ^2^ = 0.2364
β‐catenin	β = −0.6225 *p* = 0.1480 *r* ^2^ = 0.2427	–	β = −0.5065 *p* = 0.00009* *r* ^2^ = 0.8669	β = −0.324 *p* = 0.00007* *r* ^2^ = 0.8763
CB1	β = +0.8884 *p* = 0.2753 *r* ^2^ = 0.1463	β = −0.5065 *p* = 0.00009* *r* ^2^ = 0.8669	–	β = +0.6084 *p* = 0.00002* *r* ^2^ = 0.9144
CB2	β = +17,748 *p* = 0.1542 *r* ^2^ = 0.2364	β = −0.324 *p* = 0.00007* *r* ^2^ = 0.8763	β = +0.6084 *p* = 0.00002* *r* ^2^ = 0.9144	–

*Note*: The results of the analysis of regression are presented as the β coefficient, *r* ^2^ and the level of statistical significance (*p*, where *p < 0.05).

**TABLE 3:** Correlation analysis between the tested proteins in the cortex and medulla of rats with secondary hypertension.

Renovascular hypertension – Adrenal cortex				
	CACYBP/SIP	β‐catenin	CB1	CB2
CACYBP/SIP	–	β = +0.7473 *p* = 0.1497 *r* ^2^ = 0.1117	β = −1.2167 *p* = 0.0888 *r* ^2^ = 0.1524	β = +0.4039 *p* = 0.3725 *r* ^2^ = 0.0444
β‐catenin	β = +0.7473 *p* = 0.1497 *r* ^2^ = 0.1117	–	β = +0.4633 *p* = 0.1523 *r* ^2^ = 0.1104	β = +0.784 *p* = 0.00000* *r* ^2^ = 0.8363
CB1	β = −1.2167 *p* = 0.0888 *r* ^2^ = 0.1524	β = +0.4633 *p* = 0.1523 *r* ^2^ = 0.1104	–	β = +0.1622 *p* = 0.2611 *r* ^2^ = 0.0696
CB2	β = +0.4039 *p* = 0.3725 *r* ^2^ = 0.0444	β = +0.784 *p* = 0.00000* *r* ^2^ = 0.8363	β = +0.1622 *p* = 0.2611 *r* ^2^ = 0.0696	–
**Renovascular hypertension – Adrenal medulla**				
	**CACYBP/SIP**	**β‐catenin**	**CB1**	**CB2**
CACYBP/SIP	–	β = +0.9374 *p* = 0.2085 *r* ^2^ = 0.1896	β = +1.5765 *p* = 0.1523 *r* ^2^ = 0.2383	β = −1.0873 *p* = 0.3213 *r* ^2^ = 0.1226
β‐catenin	β = +0.9374 *p* = 0.2085 *r* ^2^ = 0.1896	–	β = +1.4113 *p* = 0.00005* *r* ^2^ = 0.8852	β = −1.3899 *p* = 0.00001* *r* ^2^ = 0.9285
CB1	β = +1.5765 *p* = 0.1523 *r* ^2^ = 0.2383	β = +1.4113 *p* = 0.00005 * *r* ^2^ = 0.8852	–	β = −0.9193 *p* = 0.00002* *r* ^2^ = 0.9140
CB2	β = −1.0873 *p* = 0.3213 *r* ^2^ = 0.1226	β = −1.3899 *p* = 0.00001* *r* ^2^ = 0.9285	β = −0.9193 *p* = 0.00002* *r* ^2^ = 0.9140	–

*Note*: The results of the analysis of regression are presented as the β coefficient, *r* ^2^ and the level of statistical significance (*p*, where *p < 0.05).

## 4 DISCUSSION

The pathogenesis of arterial hypertension is complex and results from disorders of many organs, including those belonging to the endocrine system. Experimental studies on animals allowed to broaden the knowledge and understanding of the mechanisms of various pathological states occurring in humans.^14^

Calcium homeostasis is subjected to complex regulation by calcium‐binding proteins. Among them, more and more attention is paid to the recently discovered CacyBP/SIP protein. This protein is responsible for the proper course of many biological processes.^15^ Among other things, it takes part in the processes of dephosphorylation and ubiquitination in the cell. CacyBP/SIP forms, together with other proteins (Siah‐1, Skp1 and the TBL1 protein), a new ubiquitin ligase complex that recognises the non‐phosphorylated form of β‐catenin. It has been suggested that β‐catenin is essential for the functioning of the cardiovascular system. By regulating the level of β‐catenin, the CacyBP/SIP protein might significantly influence cardiovascular homeostasis.^4^, ^16^ CacyBP/SIP occurs mainly in the cytoplasm, but as a result, the increased concentration of Ca^2+^ ions is transferred to the cell nucleus.^17^ The intracellular calcium content is regulated by cannabinoids that modulate the flow of calcium ions through the membrane channels.^10^ Endocannabinoids, acting through CB1 and CB2 receptors, regulate blood pressure, heart rate and contractility of the heart muscle.^18^

Considering the key importance of the above parameters in the functioning of the cardiovascular system and in the view of the close relationship between CacyBP/SIP, β‐catenin and cannabinoid receptors, we decided to compare and evaluate the gene expression and distribution of CacyBP/SIP, β‐catenin, CB1 and CB2 in the adrenal glands of rats with arterial hypertension of various aetiologies.

The current report shows that the expression of tested parameters in the adrenals of rats is altered in a state of elevated blood pressure and the intensity of these changes depends on the aetiology of arterial hypertension.

In our studies, we have demonstrated lower immunoreactivity and decreased expression of the CacyBP/SIP gene in the adrenal glands of all hypertensive rats compared to the rats of the corresponding control group. Previous studies have shown a change in CacyBP/SIP levels in various pathological states, which indicates the role of this protein in maintaining cell homeostasis.^4^, ^15^ CacyBP/SIP protects other proteins, securing them against denaturation and other degradation processes, thus protecting cells against stress factors.^19^ Inflammatory processes and oxidative stress play an important role in the pathogenesis and progression of hypertension, leading to organ complications. Experimental studies showed infiltration of lymphocytes and macrophages in various organs of animals with hypertension.^20^ In our recent study, conducted in rats with primary hypertension (SHR) and secondary hypertension (2K1C), the highest influx of immune cells in the heart of SHR rats was observed compared to other models of hypertension.^21^

There is only one report in the available literature on the role of CacyBP/SIP in the adrenal glands. This is our previous study on the relationship between CacyBP/SIP protein and the MAPK pathway in hypertension of various aetiologies. In that study, we also showed decreased expression of CacyBP/SIP in the adrenal glands of rats with primary and secondary hypertension, which could be related to oxidative stress, the role of which in the pathogenesis of hypertension has not yet been clarified. Therefore, further studies on hypertension should be conducted to clarify the impaired balance between the antioxidant system and free radicals.^22^

Reports on the role of this protein in the functioning of the cardiovascular system seem interesting. The authors provided evidence that CacyBP/SIP plays an important role in cardiomyocyte differentiation and heart development.^4^ An increase in the level of CacyBP/SIP during myocardial infarction has also been demonstrated, which suggests a protective role of this protein for cardiomyocytes against hypoxia/reoxygenation.^4^ In our previous studies, we also found an increased content of CacyBP/SIP in the heart of rats with arterial hypertension of various aetiologies, which suggests a role of this protein in hypertensive cardiac complications.^21^

Studies by Matsuzawa and Reed^16^ showed different levels of β‐catenin in CacyBP/SIP transfected cells, confirming the role of this protein in ubiquitination and degradation of β‐catenin, which is a central component of the Wnt signalling pathway and plays a key role in regulating many cellular processes.

The studies of the present work showed a decrease in the level of β‐catenin in the adrenal glands of 2K1C rats compared to the control groups, while in SHR rats no immune‐expression of β‐catenin was detected. Detailed analysis of the RT‐qPCR confirmed the results of immunohistochemical studies. This may be due to dysregulation of the mechanisms of β‐catenin and CacyBP/SIP dependence and protein degradation in a state of elevated blood pressure. Our previous studies showed similar results of the decrease in the content of β‐catenin in the adrenal glands and heart of rats in the same models of arterial hypertension.^21^, ^23^ On the other hand, Xiao et al.^24^ showed upregulated renal β‐catenin in two models of hypertension (induced by angiotensin II infusion and nephrectomy) and decrease of the blood pressure due to inhibition of Wnt/β‐catenin, which, according to these authors, indicates that overactive Wnt/β‐catenin promotes hypertension. In one of our recent studies regarding canonical Wnt signalling, we also observed a marked increase in the intensity of β‐catenin immunoreactivity in the kidneys of rats with spontaneous and renovascular hypertension.^25^ It should be stated that the kidneys and adrenal glands have different physiological functions and are under the control of different mechanisms. Also, it should be remembered that the relationship between blood pressure regulation and Wnt is bidirectional. Wnt signalling is characterised by pleiotropic effects on various pathways related to the physiology of the cardiac, renal and neural physiology, while hypertension is a heterogeneous disease entity with unique molecular pathways regulating the response of this disease in therapy.^25^

Literature data indicate an important role of the endocannabinoid system in the blood pressure regulation.^26^, ^27^ In the studies of the present work, no presence of CB1 receptors was found in the adrenal glands of SHR rats. While in the rats with 2K1C hypertension, the intensity of the immune reaction showing CB1 was weakened compared to animals from the corresponding control group. After detailed analysis of the RT‐qPCR results, significant decrease in the expression of the gene encoding CB1 gene in the adrenal glands of SHR rats compared to the control group was shown. Also, expression of the CB1 in the 2K1C rats was lower compared to the respective normotensive rats.

Ziegler et al.^11^ on human NCI‐H295R adrenal cortex cells and normal human adrenal glands demonstrated a relationship between the expression of endocannabinoid receptors and adrenal function. The authors of the cited study, demonstrating the presence of the CB1 receptor and the lack of CB2 expression in the cells of the adrenal cortex that produce angiotensin II‐responsive steroids, suggest the effect of endocannabinoids on aldosterone secretion and thus on blood pressure regulation.

The active participation of the endocannabinoid system has been confirmed in various types of hypertension.^26^, ^27^ Elevated levels of CB1 in the cardiac and vascular endothelium or blocking the metabolic degradation of anandamide regulate blood pressure. Batkai et al.^27^ showed that CB1 antagonists did not affect blood pressure in rats with normal pressure and that FAAH (fatty acid amide hydrolase) or anandamide transport inhibitors were ineffective, indicating that the endocannabinoid system is not active at normal pressure. Elevated basal blood pressure is itself responsible for the tonic activation of CB1, as evidenced by the fact that CB1 antagonists induce a sustained increase in blood pressure in rats with three different types of hypertension. Rats with spontaneous arterial hypertension showed significantly higher expression of CB1 in the heart and aortic endothelium compared to WKY animals.^27^ This proves that in hypertension, the endocannabinoid system is altered. On the other hand, Biernacki et al.^28^ showed a decrease in CB1 expression in the liver of rats with spontaneous hypertension.

The studies conducted so far have shown a significant relationship between the activity of the hypothalamic–pituitary–adrenal axis and the hormonal response to the expression of the CB1 receptor. CB1 cannabinoid receptor signalling can both inhibit and enhance the interdependence and activity of individual structures.^29^ Undoubted, the role of endocannabinoids and cannabinoid receptors in the regulation of the circulatory system in arterial hypertension takes place through complex mechanisms involving direct and indirect influence on the heart muscle and blood vessels.^27^

The significant weakening of the immunoreactivity and expression of CB1 in the adrenal glands of 2K1C rats, as well as the negative response to the presence of CB1 in SHRs observed in our studies, may be caused by dysfunction of the sympathetic‐adrenal system, participating in the pathogenesis of hypertension. However, it cannot be ruled out that the lack of detectable CB1 receptors in the adrenal gland indicates that the observed functional effects are mediated by an as yet unidentified cannabinoid receptor subtype (CBx)?

Our studies showed a significant weakening of the immunoreactivity of the CB2 receptor in the adrenal glands of the 2K1C rats, while in SHR the intensity of the reaction was clearly increased compared to the corresponding control group. The above results were confirmed by RT‐qPCR analysis of gene expression.

Perhaps the results of our research can be explained by the different intensity of oxygen‐free radicals (OFR) production in primary and secondary hypertension. Recent studies have documented the association of OFR with the increased activity of the renin‐angiotensin‐aldosterone system (RAA) in hypertension. Angiotensin II has been shown to increase NADH oxidase activity in endothelial cells and myocytes, inducing oxidative stress in the blood vessel wall.^30^ Primary hypertension is associated with an increase in the activity of the sympathetic nervous system. Although the oxidation of catecholamines is associated with the release of O_2_, experiments with norepinephrine infusion causing hypertension in rats did not show an increased amount of O_2_. An important and relatively new concept is that inflammatory cells contribute to hypertension in a OFR‐dependent fashion.^31^

To date, studies have shown various interactions between elements of the endocannabinoid system and OFR in various organs. In experimental myocardial ischemia and reperfusion, activation of peripheral CB1 receptors has been shown to promote oxidative stress, while activation of CB2 receptors reduces oxidative stress in mice and rats.^32^, ^33^ Research by Biernacki et al.^34^ showed increased lipid peroxidation and oxidative stress as well as different expression of CB1 and CB2 receptors in the heart of rats with essential and secondary hypertension.

In summary, this study shows, for the first time, marked differences in the expression of tested parameters in the adrenals of rats with primary and secondary hypertension. The current study can be considered a pilot study that may point the way for further detailed research leading to a better understanding of the complex physiology of the adrenal glands and their function in hypertension.

## AUTHOR CONTRIBUTIONS

**Magdalena Smereczańska:** Conceptualization (lead); funding acquisition (lead); investigation (lead); methodology (equal); resources (lead); writing – original draft (lead). **Natalia Domian:** Methodology (equal); resources (supporting); writing – original draft (supporting). **Alicja Lewandowska:** Formal analysis (lead); investigation (supporting); methodology (supporting). **Irena Kasacka:** Conceptualization (supporting); formal analysis (supporting); funding acquisition (supporting); investigation (supporting); methodology (supporting); resources (supporting); supervision (lead); writing – original draft (supporting); writing – review and editing (lead).

## FUNDING INFORMATION

This research was funded by Medical University of Białystok.

## CONFLICT OF INTEREST STATEMENT

The authors declare that there are no conflicts of interest.

## Data Availability

Data from this study are available from the corresponding author upon reasonable request.
